# Using linked administrative data to build a Social Disparities Index for Covid-19 to evaluate the effects of inequalities on pandemic in Brazil.

**DOI:** 10.23889/ijpds.v7i3.2088

**Published:** 2022-08-25

**Authors:** Maria Yury Travassos Ichihara, Rosemeire Leovigildo Fiaccone, Lilia Carolina Carneiro da Costa, Aline Grimberg Pereira de Medeiros, Elzo Pereira Pinto, Rafael Felipe da Silva Souza, Mauricio Lima Barreto, Anderson Ara, Emmanuelle Goes, Julia M Pescarini, Marcos Ennes Barreto, Jaime Gregorio Bellido

**Affiliations:** 1 Centre for Data and Knowledge Integration for Health (CIDACS), Oswaldo Cruz Foundation; 2 The London School of Hygiene & Tropical Medicine; 3 London School of Economics

## Objectives

Brazil is one of the most unequal countries in the world. We aimed to create a Social Disparities Index for Covid-19 (SDI-Covid-19) using linked administrative data to understand the role of inequalities in the COVID-19 pandemic to target social and health policies.

## Approach

Using linked administrative data (2010 Census and National Registry of Health Establishments) we selected municipal indicators for three domains: a) Brazilian Deprivation Index; b) % black/brown/indigenous, % people living in housing with density >2 person/room, % poor older people as sociodemographic; c) ICU beds and respirators rates weighted by distance to the health region headquarters as access to health service. A z-score weighted by the population estimated each indicator within the domains. The domains were combined by simple average of the z-scores and classified in quintiles (1-very low to 5-very high). Other methods (TRI, Macharia et al)1 found similar estimative.

## Results

The SDI-COVID-19 estimated for the three moments of the pandemic (before (T0), first (T1) and second peak (T2)) in Brazil showed that municipalities belonging to the North and Northeast regions had worse SDI-Covid-19 compared to municipalities in the Midwest, Southeast, and South regions of the country. More than 97% of the municipalities in the North and Northeast regions had high and very high SDI in the three estimated periods. There was an increase of 46% in the number of municipalities in the Midwest region with high and very high SDI in T1 and 33% in T2 when compared to T0. Otherwise, we observed an increase of municipalities with very low and low SDI in T1 and T2 in the Southeast and South regions.

## Conclusion

The deterministic linkage of administrative data allowed to build the SDI-COVID-19. This index identified the most vulnerable municipalities in the North and Northeast regions in contrast of municipalities in the Southeast and South regions of Brazil with lower inequalities during the pandemic. Findings can target policies to those areas.

